# Choosing Wisely internationally – helpful recommendations for antimicrobial stewardship!

**DOI:** 10.1007/s15010-023-02005-y

**Published:** 2023-02-25

**Authors:** Norma Jung, Lukas Tometten, Rika Draenert

**Affiliations:** 1 Department I of Internal Medicine, Division of Infectious Diseases, University of Cologne, Faculty of Medicine, University Clinics, Cologne, Germany; 2 Interdisciplinary Antibiotic Stewardship Team, LMU Klinikum, Munich, Germany

**Keywords:** Choosing wisely, Antimicrobial stewardship, Antimicrobial resistance, Pretest probability, Indication, Antibiotic overuse

## Abstract

**Purpose:**

Antimicrobial resistance poses a major threat to human health globally and antibiotic overuse is a main driver of resistance. Antimicrobial stewardship (AMS) was developed to improve the rationale use of antibiotics. The Choosing Wisely campaign was initiated to ameliorate medical practice through avoidance of unnecessary diagnostic and therapeutic procedures. Our objective was to give an overview on the Choosing Wisely recommendations related to AMS practices from a selection of different countries in order to define future needs.

**Methods:**

We evaluated the seven countries already analyzed for Choosing Wisely recommendations related to topics of infectious medicine before. Finally, we included five of the former countries (Australia/New Zealand, Canada, Italy, Switzerland, and USA) and Germany with easily accessible recommendations and selected those related to six categories of AMS as following: diagnostics, indication, choice of antiinfective drugs, dosing, application and duration of therapy.

**Results:**

In total, 213 recommendations could be extracted related to AMS for the six countries and were matched to the chosen categories. Interestingly, no recommendations were found for the category “dosing.” Topics related to indication and diagnostics were most frequently found with 85 and 78 recommendations, respectively. Perioperative prophylaxis was a frequently addressed issue – both related to application, indication and duration. Avoiding antibiotic treatment of asymptomatic bacteriuria and upper respiratory tract infections were central topics of all countries.

**Conclusion:**

AMS is an important strategy to fight increasing resistance and is frequently addressed by Choosing Wisely recommendations of different countries. Similar issues are considered important in the selected countries.

## Introduction

Antimicrobial resistance poses a major threat to human health globally and antibiotic overuse is a main driver of resistance [1, 2]. It is estimated that drug-resistant infections will increase dramatically in the coming decades without interventions [3]. WHO and other groups agree that a global action is necessary [4].

Antimicrobial stewardship (AMS) has been developed to improve the rationale use of antibiotics and guidelines related to its implementation have been published in several countries [5, 6].

The Choosing Wisely campaign – initiated in 2012 in the USA—was started to ameliorate medical practice through avoidance of unnecessary diagnostic and therapeutic procedures and has emerged nowadays in over 25 countries [7, 8]. Many multinational Choosing Wisely recommendations are related to the overuse of antibiotics and in Canada a campaign “Using antibiotics wisely” for the primary sector was implemented [9]. Nevertheless, a comprehensive review of Choosing Wisely recommendations related AMS strategies is missing.

Our objective was to give an overview on Choosing Wisely recommendations related to AMS practices of a selection of different medical societies and countries. Hereby, we adapted our search to the different AMS categories as described by the guidelines for AMS rounds, namely diagnostics, indication, application, duration, choice of drug and dosing [6].

## Methods

For this narrative review, it was decided to relate to the review written in 2015 [10]. The seven countries (Australia/New Zealand, Canada, Italy, Japan, The Netherlands, Switzerland and USA) evaluated in 2015 were chosen again. All Choosing Wisely recommendations of the seven countries and Germany were searched for those related to antimicrobial stewardship and a rational use of antiinfective agents. After the initial evaluation, however, Japan and the Netherlands had to be excluded as there were no clear recommendations to be found (Japan) or there was no collection of all recommendations which was easily accessible (Netherlands).

According to the German AWMF guideline antimicrobial stewardship, there are six categories for the rational use of antimicrobial agents during ward rounds: diagnostics, indication, choice of antiinfective drugs, dosing, application and duration of therapy [6]. We, therefore, sought to classify the international Choosing Wisely recommendations as well as the German recommendations according to these keywords.

## Results

In total, 213 Choosing Wisely recommendations concerning AMS of the six countries could be listed for this review (see Tables 1, 2, 3, 4, and 5). They were derived from 85 different societies and associations from Australia/New Zealand, Canada, Germany, Italy, Switzerland and the USA. In each country, a substantial part of the recommendations was released by non-infectious diseases (non-ID) societies. Interestingly, Germany is the only country with recommendations made only by the internal medicine societies.

**Table 1:** Recommendations on diagnostics

Topic	Recommendation	Society	Country
**General diagnostics**			
**Specimen collection**	Avoid blood cultures in patients who are not systemically septic, have a clear source of infection and in whom a direct specimen for culture (e.g. urine, wound swab, sputum, cerebrospinal fluid, or joint aspirate) is possible	Australasian College for Emergency Medicine	Australia and New Zealand
	Do not perform cultures (e.g. urine, blood, sputum cultures) or test for C. difficile unless patients have signs or symptoms of infection. Tests can be falsely positive leading to over diagnosis and overtreatment**	Society for Healthcare Epidemiology of America	United States of America
	Do not routinely obtain swabs during surgical procedures when fluid and/or tissue samples can be collected*	Association of Medical Microbiology and Infectious Diseases Canada	Canada
		American Society for Clinical Pathology	United States of America
	In patients with the clinical picture of severe bacterial infection, antibiotics should be administered rapidly after sample assay and the regimen should be reevaluated regularly**	German Society for Infectious Diseases (DGI)	Germany
	In patients with suspected severe infections, at least two pairs of blood cultures should be taken regardless of body temperature at separate puncture sites before antibiotics are administered. It is not required to maintain a minimum time interval between the sampling of the blood cultures**	German Society for Internal Medicine (DGIM)	Germany
Laboratory testing	Do not order IgM antibody serologic studies to assess for acute infection with infectious agents no longer endemic in the US, and in general avoid using IgM antibody serologies to test for acute infection in the absence of sufficient pre-test probability	American Society for Clinical Pathology	United States of America
	Do not perform Procalcitonin testing without an established, evidence-based protocol	American Society for Clinical Pathology	United States of America
	Do not perform maternal serologic studies for cytomegalovirus and toxoplasma as part of routine prenatal laboratory studies	Society for Maternal–Fetal Medicine	United States of America
	Do not request daily full blood counts, erythrocyte sedimentation rate (ESR) or C-reactive protein (CRP) as measures of response to antibiotic treatment if patients are clinically improving	Internal Medicine Society of Australia and New Zealand	Australia and New Zealand
**Anatomical entities**			
Cardiac	Do not request routinely extended incubation of blood cultures in suspected endocarditis	American Society for Microbiology	United States of America
Cerebral	Adult patients with suspected bacterial meningitis should be given dexamethasone and antibiotics after blood culture collection and prior to imaging**	German Society for Internal Medicine (DGIM)	Germany
	In suspected meningitis, a CT scan should not be performed prior to lumbar puncture—except in cases of symptoms, indicative of increased intracranial pressure or focal pathology, or in the presence of intense immunosuppression	German Society for Internal Medicine (DGIM)	Germany
	Do not routinely order nucleic acid amplification testing on cerebrospinal fluid (e.g., herpes simplex virus, varicella zoster virus, enteroviruses) in patients without a compatible clinical syndrome	Association of Medical Microbiology and Infectious Diseases Canada	Canada
	In a patient with fatigue, avoid performing multiple serological investigations, without a clinical indication or relevant epidemiology	Australasian Society for Infectious Diseases	Australia and New Zealand
Ear, Nose, Throat	Do not routinely obtain radiographic imaging for patients who meet diagnostic criteria for uncomplicated acute rhinosinusitis*	Royal Australasian College of Surgeons	Australia and New Zealand
		Canadian Society of Otolaryngology—Head and Neck Surgery	Canada
		Canadian Society of Allergy and Clinical Immunology	Canada
		Swiss Society of Oto-Rhino-Laryngology and Head and Neck Surgery (SGORL)	Switzerland
		American Academy of Allergy, Asthma and Immunology	United States of America
		American Academy of Otolaryngology—Head and Neck Surgery Foundation	United States of America
	Do not order more than one computerized tomography (CT) scan of the paranasal sinuses within 90 days to evaluate uncomplicated chronic rhinosinusitis patients when the paranasal sinus CT obtained is of adequate quality and resolution to be interpreted by the clinician and used for clinical decision-making and/or surgical planning	American Academy of Otolaryngology—Head and Neck Surgery Foundation	United States of America
	Do not swab the nasal cavity as part of the work up for rhinosinusitis	Canadian Society of Otolaryngology—Head and Neck Surgery	Canada
Gastrointestinal	Do not investigate or treat for fecal pathogens in the absence of diarrhea or other gastro-intestinal symptoms**	Australasian Society for Infectious Diseases	Australia and New Zealand
	Do not routinely test for community gastrointestinal stool pathogens in hospitalized patients who develop diarrhea after day 3 of hospitalization	American Society for Clinical Pathology	United States of America
Neutropenia	In patients with neutropenic fever (neutrophils < 0.5 G/L or < 1 G/L with a decreasing tendency), empiric therapy with broad-spectrum antibiotics should be started after taking 2 independent blood cultures and without delay due to further diagnostics**	German Society for Internal Medicine (DGIM)	Germany
Osteoarticular	Any unclear acute joint swelling should be clarified immediately by joint puncture and punctate examination	German Society for Rheumatology (DGRh)	Germany
	Do not routinely repeat radiologic imaging in patients with osteomyelitis demonstrating clinical improvement following adequate antimicrobial therapy	Association of Medical Microbiology and Infectious Diseases Canada	Canada
	Do not routinely use MRI to diagnose bone infection (osteomyelitis) in the foot	American Podiatric Medical Association	United States of America
Respiratory Tract	Do not order chest X-rays in patients with acute upper respiratory tract infections*	The Royal Australian College of General Practitioners	Australia and New Zealand
		Nurse Practitioner Association of Canada	Canada
	Do not send unnecessary or improperly collected specimens for testing	Canadian Nurses Association, Infection Prevention and Control Canada	Canada
Skin	Do not routinely use microbiologic testing in the evaluation and management of acne	American Academy of Dermatology	United States of America
	Do not routinely swab open wounds and do not prescribe systemic antibiotics based on these results, without clinical features of local or systemic infection*/**	Australasian Society for Infectious Diseases	Australia and New Zealand
		Association of Medical Microbiology and Infectious Diseases Canada	Canada
		Burns Canada	Canada
		American Podiatric Medical Association	United States of America
	Avoid wound cultures in emergency department patients with uncomplicated skin and soft tissue abscesses after successful incision and drainage and with adequate medical follow-up	American College of Emergency Physicians	United States of America
Urinary tract	Urine culture should not be carried out either routinely or in the absence of the typical symptoms of a urinary tract infection unless they are pregnant or undergoing genitourinary instrumentation where mucosal bleeding is expected; bag urine collection should be avoided*	The Royal College of Pathologists of Australasia	Australia and New Zealand
		Canadian Nurses Association, Infection Prevention and Control Canada	Canada
		Long Term Care Medical Directors Association of Canada	Canada
		Association of Medical Microbiology and Infectious Disease Canada	Canada
		Canadian Association of Pathologists	Canada
		Italian Multidisciplinary Society for the Prevention of Infections in Healthcare Organizations	Italy
		American Society for Microbiology	United States of America
	Avoid presumptive antibiotic treatment of recurrent UTIs in women without first obtaining a UA C&S (urine analysis, culture and sensitivity)**	American Urogynecologic Society	United States of America
	Do not send urine specimens for culture on asymptomatic patients including the elderly, diabetics, or as a follow up to confirm effective treatment	Canadian Association of Pathologists	Canada
**Pathogens**			
*Borrelia burgdorferi*	Avoid serologic testing of Borreliosis in patients without specific symptoms*	German Society for Rheumatology (DGRh)	Germany
		Swiss Society for Infectious Diseases	Switzerland
		Swiss Society for Rheumatology	Switzerland
		American College of Rheumatology	United States of America
	Do not order Lyme serology on patients with a primary erythema migrans lesion	American Society for Microbiology	United States of America
*Chlamydia* spp.	Do not screen for chlamydia using serological tests	Australasian Chapter of Sexual Health Medicine	Australia and New Zealand
*Chlamydia trachomatis/Neisseria gonorrhoeae*	Do not routinely send urine for Chlamydia trachomatis and Neisseria gonorrhoeae (CT/NG) testing from females if vaginal swab collection is possible	American Society for Microbiology, American Society for Clinical Laboratory Science & American Society for Clinical Pathology	United States of America
*Clostridioides difficile*	Do not routinely collect or process specimens for Clostridium difficile testing when stool is non-liquid (i.e., does not take the shape of the specimen container) or when the patient has had a prior nucleic acid amplification test result within the past 7 days*	Association of Medical Microbiology and Infectious Disease Canada	Canada
		Canadian Nurses Association, Infection Prevention and Control Canada	Canada
		Swiss Society for Infectious Diseases	Switzerland
		American Society for Microbiology	United States of America
		Society for Healthcare Epidemiology of America	United States of America
		Infectious Diseases Society of America	United States of America
	Do not obtain a C. difficile toxin test to confirm “cure” if symptoms have resolved	The Society for Post-Acute and Long-Term Care Medicine	United States of America
*Helicobacter pylori*	Do not request serology for H. pylori. Use the stool antigen or breath tests instead	American Society for Clinical Pathology	United States of America
Hepatitis C virus	Do not repeat Hepatitis C virus antibody testing in patients with a previous positive Hepatitis C virus (HCV) test. Instead, order Hepatitis C viral load testing for assessment of active versus resolved infection	American Society for Clinical Pathology	United States of America
	Do not repeat Hepatitis C viral load testing in an individual who has established chronic infection, outside of antiviral treatment*	Canadian Association for the Study of Liver Disease	Canada
		American Association for the Study of Liver Diseases	United States of America
Herpes simplex virus	Do not order herpes serology unless there is a clear clinical indication*	Australasian Chapter of Sexual Health Medicine	Australia and New Zealand
		Swiss Society for Dermatology and Venerology (SGDV)	Switzerland
		American Academy of Family Physicians	United States of America
	Do not use herpes simplex virus (HSV) polymerase chain reaction (PCR) testing for genital HSV infection screening in adults and adolescents. Real-time HSV PCR testing should only be used to confirm herpes diagnosis in patients with suspected herpes	American Society for Clinical Laboratory Science	United States of America
HIV	Avoid quarterly viral load testing of patients who have durable viral suppression, unless clinically indicated	HIV Medicine Association	United States of America
	Do not routinely repeat CD4 measurements in patients with HIV infection with HIV-1 RNA suppression for ≥ 2 years and CD4 counts ≥ 500/µL, unless virologic failure occurs or intercurrent opportunistic infection develops	Association of Medical Microbiology and Infectious Disease Canada	Canada
	Avoid unnecessary CD4 tests	HIV Medicine Association	United States of America
	Do not order complex lymphocyte panels when ordering CD4 counts	HIV Medicine Association	United States of America
	Do not routinely test for CMV IgG in HIV-infected patients who have a high likelihood of being infected with CMV	HIV Medicine Association	United States of America
Influenza virus	Do not test for influenza unless the patient is symptomatic and the result will influence clinical management and decision making	American Society for Microbiology, American Society for Clinical Laboratory Science & American Society for Clinical Pathology	United States of America
Group B* Streptococcus*	Do not perform 3rd trimester Group B streptococcus (GBS) culture in patients with GBS bacteriuria during pregnancy	Society for Maternal–Fetal Medicine	United States of America
Mycobacterium tuberculosis	Do not do unnecessary screening tuberculin skin tests (TSTs)	Public Health Physicians of Canada	Canada
*Ureaplama spp.*	Do not test for ureaplasma species in asymptomatic patients	Australasian Chapter of Sexual Health Medicine	Australia and New Zealand

*For the exact wording of the Societies' recommendations, refer to the original recommendations

**Recommendation listed in two or more tables

**Table 2:** Recommendations on indications

Topic	Recommendation	Society	Country
**General indication**			
	Do not initiate an antibiotic without an identified indication and a predetermined length of treatment or review date*	The Society of Hospital Pharmacists of Australia	Australia and New Zealand
		Society for Healthcare Epidemiology of America	United States of America
		Society of Critical Care Medicine	United States of America
	Do not prescribe antibiotics or opioid analgesics without an examination	The Canadian Association of Hospital Dentists	Canada
	Do not routinely suggest antimicrobial treatment for older persons unless they are consistent with their goals of care	Canadian Nurses Association -	Canada
		Canadian Gerontological Nursing Association	
	Do not treat an elevated C‑reactive protein (CRP) or procalcitonin in serum with antibiotics for patients not presenting signs or symptoms of infection	German Society for Infectious Diseases (DGI)	Germany
	In severe sepsis and septic shock, calculated and high-dose antibiotic therapy should be started quickly	German Society for Infectious Diseases (DGI)	Germany
	In patients with suspected severe infections, at least two pairs of blood cultures should be taken regardless of body temperature at separate puncture sites before antibiotics are administered. It is not required to maintain a minimum time interval between the sampling of the blood cultures**	German Society for Internal Medicine (DGIM)	Germany
	Do not prescribe antibiotics to prevent infectious complications from neutropenia in cancer patients treated with standard dose chemotherapy	Italian College of Chief Hospital Medical Oncologists (CIPOMO)	Italy
**Anatomical entities**			
Cardiac	Avoid routine use of infective endocarditis prophylaxis in mild to moderate native valve disease	Italian Association of Clinical, Preventive and Rehabilitative Cardiology	Italy
	Avoid prophylactic antibiotics for the treatment of mitral valve prolapse	Infectious Diseases Society of America	United States of America
Cerebral	Adult patients with suspected bacterial meningitis should be given dexamethasone and antibiotics after blood culture collection and prior to imaging**	German Society for Internal Medicine (DGIM)	Germany
Ear, Nose, Throat	Do not prescribe oral antibiotics for uncomplicated acute external otitis	Royal Australasian College of Surgeons	Australia and New Zealand
		Swiss Society for Otorhinolaryngology, Neck and Facial Surgery	Switzerland
		American Academy of Otolaryngology—Head and Neck Surgery Foundation	United States of America
	Do not use antibiotics in adults and children with uncomplicated acute otitis media	Canadian Association of Emergency Physicians	Canada
	Do not use oral antibiotics as a first line treatment for patients with painless ear drainage associated with a tympanic membrane perforation or tympanostomy tube unless there is evidence of developing cellulitis in the external ear canal skin and pinna*	Royal Australasian College of Surgeons	Australia and New Zealand
		Canadian Society of Otolaryngology—Head & Neck Surgery	Canada
		American Academy of Otolaryngology—Head and Neck Surgery Foundation	United States of America
	Do not routinely prescribe antibiotics for acute mild-to-moderate sinusitis unless symptoms last for ten or more days, or symptoms worsen after initial clinical improvement*	Canadian Society of Allergy and Clinical Immunology	Canada
		American Academy of Asthma, Allergy and Immunology	United States of America
		American Academy of Family Physicians	United States of America
		American Academy of Sleep Medicine	United States of America
	Do not routinely use antibiotics in adults and children with uncomplicated sore throats	Canadian Association of Emergency Physicians	Canada
Eyes	Don´t use topical antibiotics for viral or nonspecific conjunctivitis*	Swiss Ophthalmological Society	Switzerland
		American Academy of Ophthalmology	United States of America
	Do not routinely provide antibiotics before or after intravitreal injections*	The Royal Australian and New Zealand College of Ophthalmologists	Australia and New Zealand
		Swiss Ophthalmological Society	Switzerland
		American Academy of Ophthalmology	United States of America
Gastrointestinal	Do not prescribe prophylactic antibiotics to prevent travellers’ diarrhea	Nurse Practitioner Association of Canada	Canada
	Do not investigate or treat for fecal pathogens in the absence of diarrhea or other gastro-intestinal symptoms**	Australasian Society for Infectious Diseases	Australia and New Zealand
Neutropenia	In patients with neutropenic fever (neutrophils < 0.5 G/L or < 1 G/L with a decreasing tendency), empiric therapy with broad-spectrum antibiotics should be started after taking 2 independent blood cultures and without delay due to further diagnostics**	German Society for Internal Medicine (DGIM)	Germany
Respiratory tract	Avoid prescribing antibiotics for upper respiratory infections*	Australasian Society for Infectious Diseases	Australia and New Zealand
		Canadian Association of Emergency Physicians	Canada
		German Society for Pneumology and Respiratory Medicine (DGP)	Germany
		German Society for Infectious Diseases (DGI)	Germany
		International Society of Doctors for the Environment	Italy
		Italian Society of General Medicine and Primary Care	Italy
		Swiss Society for General Internal Medicine	Switzerland
		Swiss Society for Infectious Diseases	Switzerland
		Infectious Diseases Society of America	United States of America
	Do not recommend antibiotics for infections that are likely viral in origin, such as an influenza-like illness	Canadian Nurses Association Infection Prevention and Control Canada	Canada
		College of Family Physicians of Canada	Canada
	Do not treat adult cough with antibiotics even if it lasts more than 1 week, unless bacterial pneumonia is suspected (mean viral cough duration is 18 days)	Canadian Thoracic Society	Canada
	Do not use antibiotics for acute asthma exacerbations without clear signs of bacterial infection	The Thoracic Society of Australia and New Zealand	Australia and New Zealand
		Canadian Thoracic Society	Canada
		Canadian Association of Emergency Physicians	Canada
Skin	Do not routinely swab open wounds and do not prescribe systemic antibiotics based on these results, without clinical features of local or systemic infection*/**	Australasian Society for Infectious Diseases	Australia and New Zealand
		Burns Canada	Canada
		Association of Medical Microbiology and Infectious Diseases Canada	Canada
		American Podiatric Medical Association	United States of America
	Do not routinely use antibiotics to treat bilateral swelling and redness of the lower leg unless there is clear evidence of infection*	Canadian Dermatology Association	Canada
		American Academy of Dermatology	United States of America
		Infectious Diseases Society of America	United States of America
	Do not routinely prescribe topical combination corticosteroid/antifungal products	Canadian Dermatology Association	Canada
	Do not routinely prescribe antibiotics for inflamed epidermoid cysts (formerly called sebaceous cysts) of the skin*	The Australasian College of Dermatologists	Australia and New Zealand
		American Academy of Dermatology	United States of America
	Monotherapy for acne with either topical or systemic antibiotics should be avoided	The Australasian College of Dermatologists	Australia and New Zealand
	Do not use oral antibiotics for acne vulgaris for more than 3 months without assessing efficacy	Canadian Dermatology Association	Canada
	Do not routinely use oral antibiotics for treatment of atopic dermatitis	American Academy of Dermatology	United States of America
	Do not administer prophylactic antibiotics to patients presenting with acute burn injuries	Burns Canada	Canada
Urinary tract	Do not prescribe antimicrobials to patients using indwelling or intermittent catheterization of the bladder unless there are signs and symptoms of urinary tract infection*	Canadian Association of Physical Medicine and Rehabilitation	Canada
		American Urological Association	United States of America
	Do not treat asymptomatic bacteriuria with antibiotics*	The Royal College of Pathologists of Australasia	Australia and New Zealand
		Australian and New Zealand Society for Geriatric Medicine	Australia and New Zealand
		Australasian Society for Infectious Diseases	Australia and New Zealand
		Canadian Urological Association	Canada
		Canadian Nurses Association	Canada
		Society of Hospital Medicine	Canada
		American Geriatrics Society	Canada
		German Society for Infectious Diseases (DGI)	Germany
		International Society of Doctors for the Environment	Italy
		Multidisciplinary Geriatrics Association	Italy
		Swiss Society for Gynecology and Obstetrics	Switzerland
		Swiss Society for Geriatrics	Switzerland
		Infectious Diseases Society of America	United States of America
		American Geriatrics Society	United States of America
	Avoid presumptive antibiotic treatment of recurrent UTIs in women without first obtaining a UA C&S (urine analysis, culture & sensitivity)**	American Urogynecologic Society	United States of America
**Pathogens**			
Multi-resistant organisms	Do not prescribe antibiotic therapy to patients colonized by multi-resistant microorganisms without signs of infection	Scientific Society of Internal Medicine	Italy
Fungi	Do not treat Candida recovered from respiratory or gastrointestinal tract specimens	German Society for Infectious Diseases (DGI)	Germany
	Do not treat recurrent or persistent symptoms of vulvovaginal candidiasis with topical and oral anti-fungal agents without further clinical and microbiological assessment	Australasian Society for Infectious Diseases	Australia and New Zealand
	Do not prescribe systemic anti-fungals for suspected onychomycosis without mycological confirmation of dermatophyte infection*	Canadian Dermatology Association	Canada
		Swiss Society for Dermatology and Venerology	Switzerland
		American Academy of Dermatology	United States of America
Group A *Streptococcus*	Do not provide antibiotic prophylaxis to all contacts of severe invasive Group A Streptococcus (iGAS) infections	Public Health Physicians of Canada	Canada

*For the exact wording of the Societies' recommendations, refer to the original recommendations

**Recommendation listed in two or more tables

**Table 3:** Recommendations on choice of antiinfective drugs

Topic	Recommendation	Society	Country
Aminoglycosides	Do not prescribe aminoglycosides for synergy to patients with bacteremia or native valve infective endocarditis caused by Staphylococcus aureus	Association of Medical Microbiology and Infectious Disease	Canada
Broad spectrum antibiotics	Do not administer broad-spectrum antibiotics without assessing the appropriateness of treatment at baseline and the possibility of de-escalation each day*	Australian and New Zealand Intensive Care Society	Australia and New Zealand
		Canadian Society of Hospital Pharmacists	Canada
		Swiss Society for Intensive Care Medicine	Switzerland
	In patients with neutropenic fever (neutrophils < 0.5 G/L or < 1 G/L with a decreasing tendency), empiric therapy with broad-spectrum antibiotics should be started after taking 2 independent blood cultures and without delay due to further diagnostics**	German Society for Internal Medicine (DGIM)	Germany
	In patients with the clinical picture of severe bacterial infection, antibiotics should be administered rapidly after sample assay and the regimen should be reevaluated regularly**	German Society for Infectious Diseases (DGI)	Germany
Cephalosporins	Oral cephalosporins should not be used for initial therapy in community-acquired pneumonia (CAP)	German Society for Pneumology and Respiratory Medicine	Germany
Drug interactions	Do not use strong CYP3A4 and P-glycoprotein inhibitors or inducers with Direct Oral Anticoagulants (DOACs) and periodically assess the medication regimen for such drug-drug interactions	American Society of Consultant Pharmacists	United States of America
	Certain opioids should not be combined with clarithromycin and other inhibitors of cytochrome 3A4	German Society for Internal Medicine (DGIM)	Germany
	Rifampicin interacts with many drugs. It should especially not be administered concomitantly with DOACs	German Society for Internal Medicine (DGIM)	Germany
	Combination therapy of citalopram/escitalopram and macrolides should not be used	German Society for Internal Medicine (DGIM)	Germany
Fluoroquinolones	Do not use fluoroquinolone antibiotics in empiric therapies, even if for severe infections, but use antibiotics with less impact on antibiotic resistance phenomenon and with less side effects*	Italian Multidisciplinary Society for Infection Prevention in Health Care Organizations	Italy
		American Urological Association	United States of America
		American Urogynecologic Society	United States of America
**Other**	In patients with pneumonia, therapy should be given according to assignment to one of the three forms (severity grades) in the emergency department	German Society for Internal Medicine (DGIM)	Germany
Penicillin Allergy	Do not overuse non-beta lactam antibiotics in patients with a history of penicillin allergy, without an appropriate evaluation	Canadian Society of Allergy and Clinical Immunology	Canada
		American Academy of Allergy, Asthma and Immunology	United States of America
	Do not prescribe alternate second-line antimicrobials to patients reporting non-severe reactions to penicillin when beta-lactams are the recommended first-line therapy	Association of Medical Microbiology and Infectious Disease	Canada

*For the exact wording of the Societies' recommendations, refer to the original recommendations

**Recommendation listed in two or more tables

**Table 4:** Recommendations on applications

Topic	Recommendation	Society	Country
Aminoglycosides	Do not give multiple daily doses of aminoglycoside antibiotics to patients with normal and stable kidney function as the risk of toxicity is less with a single daily dose	Australian and New Zealand Society of Nephrology	Australia and New Zealand
Oral Stepdown	Do not routinely prescribe intravenous forms of highly bioavailable antimicrobial agents for patients who can reliably take and absorb oral medications*	Association of Medical Microbiology and Infectious Disease	Canada
		Canadian Nurses Association	Canada
		Canadian Gerontological Nursing Association	Canada
		German Society for Infectious Diseases (DGI)	Germany
	Once patients have become afebrile (non-feverish) and are clinically improving, Do not continue prescribing intravenous antibiotics to those with uncomplicated infections and no high-risk features if they are tolerant of oral antibiotics	Internal Medicine Society of Australia and New Zealand	Australia and New Zealand
Perioperative Prophylaxis	Never administer antibiotics for perioperative prophylaxis before 60 min prior to surgical incision; the ideal time is upon induction of anesthesia	The Italian Association of Doctors of the Hospital Directions	Italy
		The Italian Society of Hygiene, Preventive Medicine and Public Health	Italy
	Never administer antibiotics for perioperative prophylaxis beyond 24 h after surgery. Antibiotic prophylaxis should be limited to the perioperative period. The choice to continue prophylaxis beyond the first 24 postoperative hours is not justified**	The Italian Association of Doctors of the Hospital Directions	Italy
		The Italian Society of Hygiene, Preventive Medicine and Public Health	Italy
	Do not continue antibiotics used for surgical prophylaxis after the patient has left the operating room*/**	German Society for Infectious Diseases (DGI)	Germany
		Swiss Society for Infectious Diseases	Switzerland
		AAGL	United States of America
		American Society for Metabolic and Bariatric Surgery	United States of America
		Society for Healthcare Epidemiology of America	United States of America
	Do not routinely use topical antibiotics on a surgical wound*	Canadian Dermatology Association	Canada
		American Academy of Dermatology	United States of America
	Don´t routinely prescribe antibiotic in patients undergoing dental extractions*	The Canadian Association of Hospital Dentists	Canada
		Italian Society of Odontostomatological Surgery	Italy
	Do not use perioperative antibiotic prophylaxis for skin procedures without additional risk factors	Swiss Society for Dermatology and Venerology	Switzerland
	Do not prescribe antibiotics after incision and drainage of uncomplicated skin abscesses unless extensive cellulitis exists	Canadian Association of Emergency Physicians	Canada
	Do not prophylactically use compounded antibiotic soaks for aftercare following office-based procedures (e.g., nail and skin lesion removal)	American Podiatric Medical Association	United States of America

*For the exact wording of the Societies' recommendations, refer to the original recommendations

**Recommendation listed in two or more tables

**Table 5:** Recommendations on duration of therapy

Topic	Recommendation	Society	Country
Discontinuation of antibiotic treatment	Do not continue antibiotics beyond 72 h in hospitalized patients unless patient has clear evidence of infection*	Society for Healthcare Epidemiology of America	United States of America
		Canadian Society of Hospital Pharmacists	Canada
	Unnecessarily long antibiotic therapy should be avoided	German Society for Internal Intensive Care and Emergency Medicine	Germany
Perioperative Prophylaxis	Never administer antibiotics for perioperative prophylaxis beyond 24 h after surgery. Antibiotic prophylaxis should be limited to the perioperative period. The choice to continue prophylaxis beyond the first 24 postoperative hours is not justified**	The Italian Association of Doctors of the Hospital Directions	Italy
		The Italian Society of Hygiene, Preventive Medicine and Public Health	Italy
	Do not continue antibiotics used for surgical prophylaxis after the patient has left the operating room*/**	German Society for Infectious Diseases (DGI)	Germany
		Swiss Society for Infectious Diseases	Switzerland
		AAGL	United States of America
		American Society for Metabolic and Bariatric Surgery	United States of America
		Society for Healthcare Epidemiology of America	United States of America

*For the exact wording of the Societies' recommendations, refer to the original recommendations

**Recommendation listed in two or more tables

Categorizing them according to their respective topic, most recommendations could be found for “indication” (*n* = 85, 40%) and “diagnostics” (*n* = 78, 37%). Issues that were addressed within the category “indication” by all six countries were avoiding antibiotic treatment in asymptomatic bacteriuria or upper respiratory infections with mostly viral origin. In addition, recommendations against antibiotics for mild-to-moderate sinusitis and against the treatment of microbiological results of superficial wound swaps were also frequently found.

Another central issue was the prophylactic use of antibiotics. In this respect, not only surgical prophylaxis was addressed but also antibiotic prophylaxis in neutropenic patients, travelers’ diarrhea, or acute burn injuries. Moreover, prophylactic use of antibiotics could also be categorized in “application” (when to give prophylactic substances if indicated) and “duration” (especially not to prolong surgical prophylaxis).

Results for “diagnostics” were much more diverse comparing the different countries. Avoiding urine cultures without symptoms of urinary tract infections was recommended most frequently within this category in four of the countries studied. This was followed by the recommendations not to test for *C. difficile* colitis in patients without diarrhea and not to obtain radiographic imaging in acute rhinosinusitis.

Much less recommendations were to be found for the other four categories. Worth mentioning for “choice of antiinfective drug” is the request to question anamnestic penicillin allergy and the cautious use of fluoroquinolones. Within “application” several countries recommend the oral use of antibiotics whenever possible. For the category “dosing”, no recommendations could be found which is interesting as the dosage of antibiotics is an important AMS topic. Consequently, there is no table for this category.

Overall, most of the recommendations advise against certain diagnostic or therapeutic measures (200/213, 94%) Germany keeps an exceptional position here: all positive recommendations listed for the review are from this country. In addition, recommendations within the six countries studied concentrate on similar fields and also gaps in recommendations are alike.

## Discussion

In light of increasing bacterial resistance globally [1], we aimed to analyze the implementation of antimicrobial stewardship goals within the simple Choosing Wisely recommendations internationally. For this review, a substantial number of recommendations concerning AMS could be listed screening six countries.

For all countries and societies assessed, it can be stated that “indication” is central in AMS for both diagnostic measures and antiinfective therapy. For “diagnostics”, an important issue found in many recommendations is the “pretest” probability, i.e., what is the likelihood for an infection in the respective patient [11]. To truly understand the result of a test and properly diagnose a patient, we must use pretest probability. In interpreting microbiological results, it is frequently stressed that a differentiation has to be made between colonization and infection and that viral infections are not to be treated with antibiotics. These are important issues that can be found in many national and international guidelines concerning AMS or certain infections [5, 6, 12]. Repeating them in the simple Choosing Wisely recommendations seemed important for all countries included in this review.

A broad consensus could also be found concerning surgical prophylaxis. The incorrect timing, false indication and prolongation – shown to be associated with acute kidney failure and *Clostridioides difficile* infection but with no reduction in the incidence of surgical site infections, seems to be a problem in all countries studied [13, 14]. Despite increasing efforts in adjusting indication and duration of perioperative prophylaxis, there is still much misuse of antibiotics after surgical procedures. A fact which most likely reflects not only a lack of knowledge but also uncertainty and concern regarding the surgical outcome [15, 16]. Here another aspect of AMS is in demand: the psychological point which extends beyond simple recommendations. A rationale use of antibiotics often implies a behavioural change [17–20].

Surprisingly, no recommendations were found concerning “dosage” – a gap that should maybe lead to the creation of new recommendations. Reasons for this might be the difficulty of a dosage specification which is dependable on many variables (kidney function, body mass index and others). In addition, the dosage of antibiotic substances is also dependent on the microbiological resistance testing with e.g. pathogens tested as “increased dosage” [21]. With regard to the continuous application of β-lactam antibiotics, recommendations are probably missing as there is still a discussion about a gap of evidence for patient-centered endpoints [22, 23].

Almost all recommendations listed here were negative ones, i.e., advising against a certain measure. This reflects the original idea of the Choosing Wisely campaign to rather avoid unnecessary interventions [8]. It also fits the AMS notion – given the current habit in antibiotic use, doing less is often the advice to clinicians in stewardship interventions [24]. Germany represents an exception with its “Klug entscheiden” initiative publishing negative and positive recommendations [25].

Another interesting point was the finding that recommendations of all six countries concerned similar topics. This is most likely due to the fact that six industrial countries were chosen with very similar socio-economic status and similar health care systems. Recommendations in low-income countries conceivably would have been different with health care problems which differ substantially to the ones seen in the countries chosen for the review. A specific guidance for setting up AMS in low- and middle-income countries is discussed [26].

This leads to a limitation of our review, the selection of the six countries. We had decided to concentrate on the countries which participated in the Choosing Wisely initiative from the beginning and were evaluated for the review published a few years ago [10]. However, in recent years many more countries began the establishment of Choosing Wisely recommendations. But an overview of all possible countries would have exceeded the scope of this study.

For the review, we did not address prophylaxis regarding central venous catheters or even more important urine catheters. This is an important AMS issue as well, but however represents a large overlap with clinical hygiene and was therefore left out.

## Conclusions

AMS is an important strategy to combat increasing antibiotic resistance. The Choosing Wiselycampaign addresses multiple topics related to AMS and might be a helpful instrument to attract attention for improving the implementation of AMS.

This work is dedicated to the 50th anniversary of *Infection*.

## Data Availability

Not applicable.
